# Progress in Aging Epidemiology in Japan: The JAGES Project

**DOI:** 10.2188/jea.JE20160093

**Published:** 2016-07-05

**Authors:** Katsunori Kondo

**Affiliations:** 1 Department of Social Preventive Medical Sciences, Center for Preventive Medical Sciences, Chiba University, Chiba, Japan; 2 Department of Gerontological Evaluation, Center for Gerontology and Social Science, National Center for Geriatrics and Gerontology, Obu, Aichi, Japan

**Keywords:** aging, gerontology, community participation, health promotion, Age Friendly Cities

## Abstract

Aging is a prominent topic in global health. The purpose of this report is to document progress in two of our research projects in Japan, which currently is the most aged society in the world. The Japan Gerontological Evaluation Study (JAGES) is one of the largest nation-wide research projects on aging, with more than 100 000 participants in 2010 and 2013. One of the notable findings is that community participation is a significant determinant of older people’s health. We have also made progress in the development of the JAGES Health Equity Assessment and Response Tools (HEART), which is a management tool for developing age-friendly cities. This progress suggests that community perspective and management of health promotion in the communities are valuable and require further research.

## INTRODUCTION

Aging is one of today’s prominent global health issues. Not only affecting developed countries, the phenomenon of the rapid aging of society is spreading in developing countries as well. WHO focused on aging and health in 2012, choosing it as the topic of world health day and publishing a special report focused on dementia.^1^ Japan is the highest ranking country in longevity and in the proportion of older people among the total population, and it is known to be the most aged society in the world. Japan is already facing many of the problems of an aging society that other countries will inevitably face in the near future. Japanese researchers have been conducting research on this topic to describe the implications of this change in society and to propose solutions to this new challenge.

This report gives a brief overview of two such projects. The first project, the Japan Gerontological Evaluation Study (JAGES), is a nationwide panel survey aiming to understand the older population in Japan. The second project is the JAGES Health Equity Assessment and Response Tool (HEART), which is a management tool developed for the older population in Japan. This article is based on a presentation at the 2015 Global Health Workshop of the Association of Pacific Rim Universities (APRU), which was hosted by Osaka University on October 30–31, 2015 in Osaka, Japan.

## JAPAN GERONTOLOGICAL EVALUATION STUDY

JAGES is one of the few population-based gerontological surveys in Japan focused on social determinants of health and social environment. It was developed from a smaller survey called the Aichi Gerontological Evaluation Survey (AGES), which was performed in two municipalities in Aichi prefecture in 1999. Four waves of questionnaire surveys were conducted in 2003–04 (15 municipalities in 3 prefectures),^2^ 2006–07 (9 municipalities in 3 prefectures), 2010–11 (31 municipalities in 12 prefectures), and 2013 (30 municipalities in 14 prefectures) for the older population (65 years and older). The project was renamed JAGES in 2010 to reflect the fact that the survey covers a wide area in Japan. The response rate for the 2003–04 wave was 55.2% (32 891 respondents), 60.8% (39 765 respondents) in 2006–07, 66.3% (112 000 respondents) in 2010–11, and 70.3% (138 000 respondents) in 2013.

JAGES aims to understand the overall status of the older population and how this population changes over time. The multi-dimensional variables covered in the surveys include health factors, psychological factors, functional factors, and social determinants of health (Table). The multiple-wave survey following the same panel over time enables discussion of causality. This report focuses on findings related to social participation and health because of their implications for promoting a healthy aging society based on evidence from JAGES.

**Table.** Factors included in the JAGES questionnaire

Healthy Aging	Social Determinants of Health
**Health**	**Socio economic status**
Comorbidity	Household type: alone/cohabitance
Smoking/alcohol	Educational attainment
BMI	Income
Teeth/oral functions	**Social role**
Fall	Employment
**Psychological**	Housework
Depression: GDS 15	Volunteer
Self-rated health	**Social Participation**
Sense of Coherence	Type of organization
**Functions**	Number of organizations
ADL; IADL	**Social Support**
	Receive/provide
	Emotional/instrumental
	Abuse
	**Social Capital**
	Perceived trust, reciprocity
	Structure: participation in community organization

Reference: Kondo K. Health Inequalities in Japan: An Empirical Study of the Older People. Melbourne: Trans Pacific Press; 2010.

## SOCIAL PARTICIPATION AND HEALTH FACTORS AS DEPICTED BY JAGES

**Figure 1.**
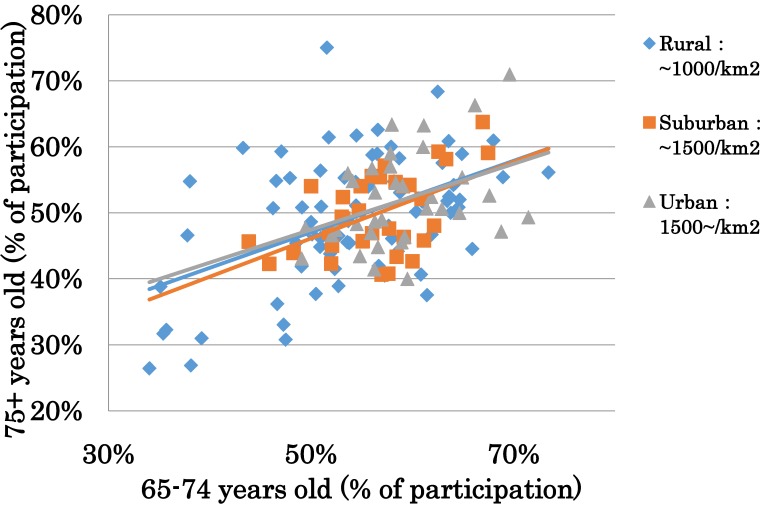
Percentage of participation by age group The figure shows percentage of participation based by age group in each school district. The different marks indicate the population density of the area and are categorized as rural, suburban, and urban.

The JAGES study looked at participation in different kinds of organizations in the community. Results from the 2010 wave show that, the older people participate in hobby clubs (36.7% of the sample), sports clubs (22.7%), senior clubs (14.6%), neighbor associations (12.6%), volunteer groups (11.4%), industrial organizations (8.8%), religious groups (8.4%), and political organizations (6.1%) at least once or twice a month. However, these percentages differed greatly between school districts (Figure 1). School districts (*n* = 141) are smaller communities within a certain municipality. Figure 1 also shows that where more young-old people (65–74 years old) participate in organizations, more old-old people (75 years old and over) participate as well, implying the importance of promoting participation among the younger generation, as these participation trends tend to continue as they age.

**Figure 2.**
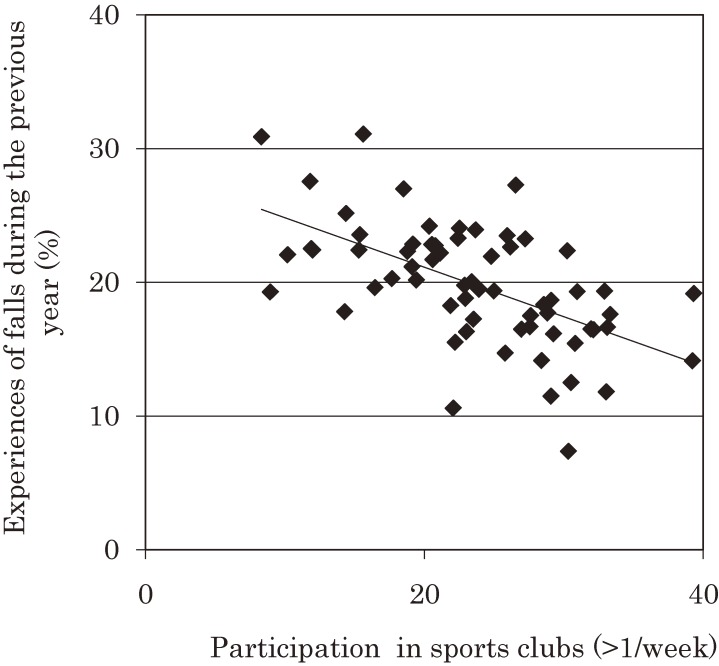
Rate of falls and participation in sports clubs The figure shows the percentage of people who experienced falls during the previous year, and participation in sports clubs more than once per week. The data only includes independent young old (65–74 years old) who do not have depression symptoms. Reference: Hayashi T, Kondo K, Yamada M, Matsunomoto D. Are there communities with less faller?—Disparity between community and relating factors: JAGES project. Kousei no Shihyo. 2014;61:1–7 (in Japanese).

The study also looked at the relationship between participation and health factors, which implied preventive effects of social participation on health. For example, the participants were asked if they fell during the previous year. The average annual rate of falling among older people in Japan is roughly 20%. However, this study revealed that the rate varied widely from 7.4% to 31.1% between different school districts (Figure 2).^3^ Similar results were found regarding the prevalence of limitation of instrumental activities of daily living (IADL), which is known to be a risk factor for dementia.^4^ Between school districts, the prevalence of limited IADL among young-old people also varied widely from 7.9% to 23.2% between school districts.

**Figure 3.**
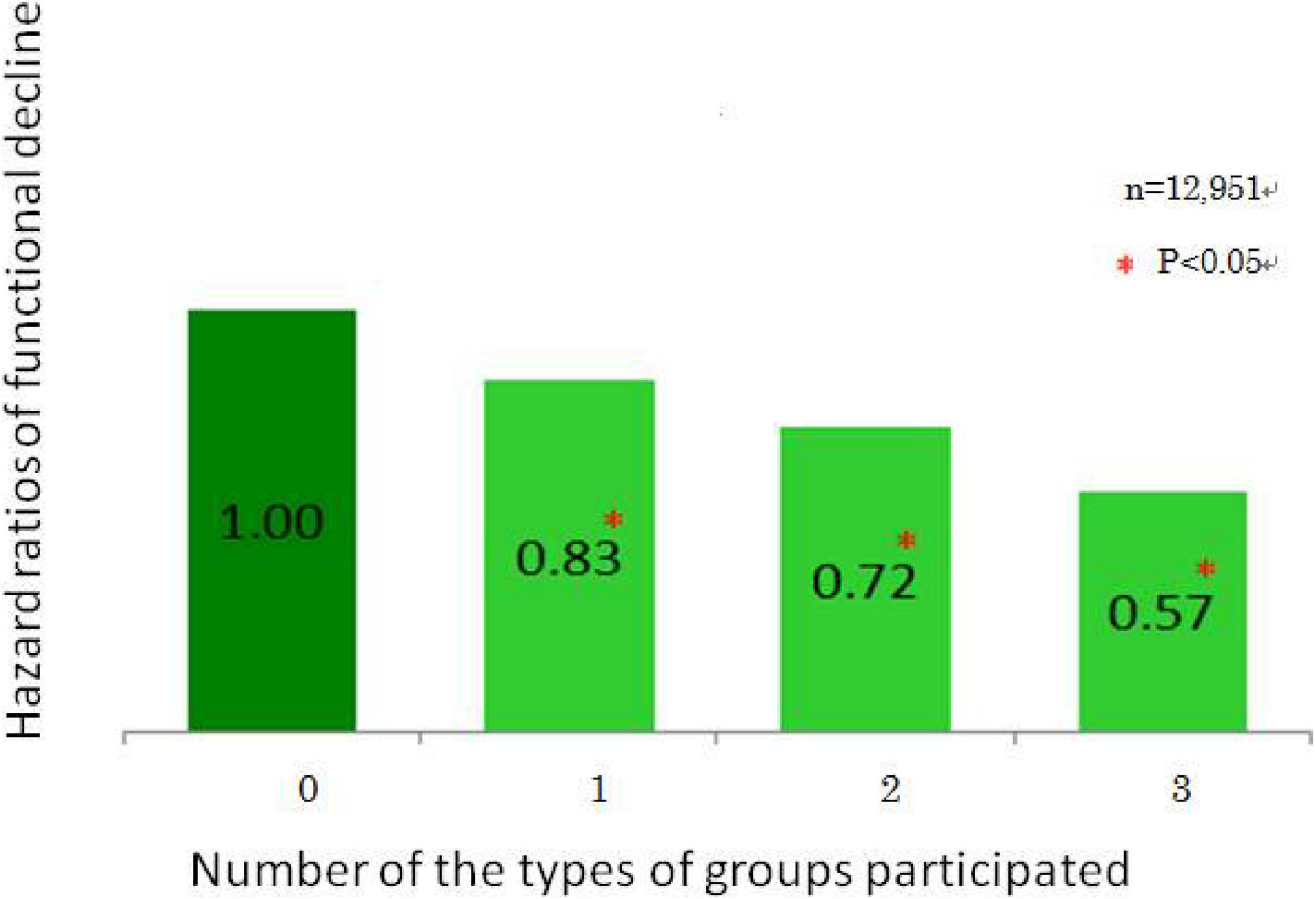
Hazard ratios of functional decline by the number of the types of groups in which subjects participated: 4-year follow-up This figure shows the hazard ratios of functional decline by the number of types of groups in which subjects participated. Hazard ratios were calculated after the 4-year follow-up and are adjusted for age, sex, number of diseases, income, education, marital status, and employment status. Reference: Kanamori S, Kai Y, Aida J, Kondo K, Kawachi K, Hirai H, Shirai K, Ishikawa Y, Suzuki S; the JAGES group. Social participation and the prevention of functional disability in older Japanese: the AGES Cohort Study. PLoS One. 2014. URL: http://www.plosone.org/article/info%3Adoi%2F10.1371%2Fjournal.pone.0099638

**Figure 4.**
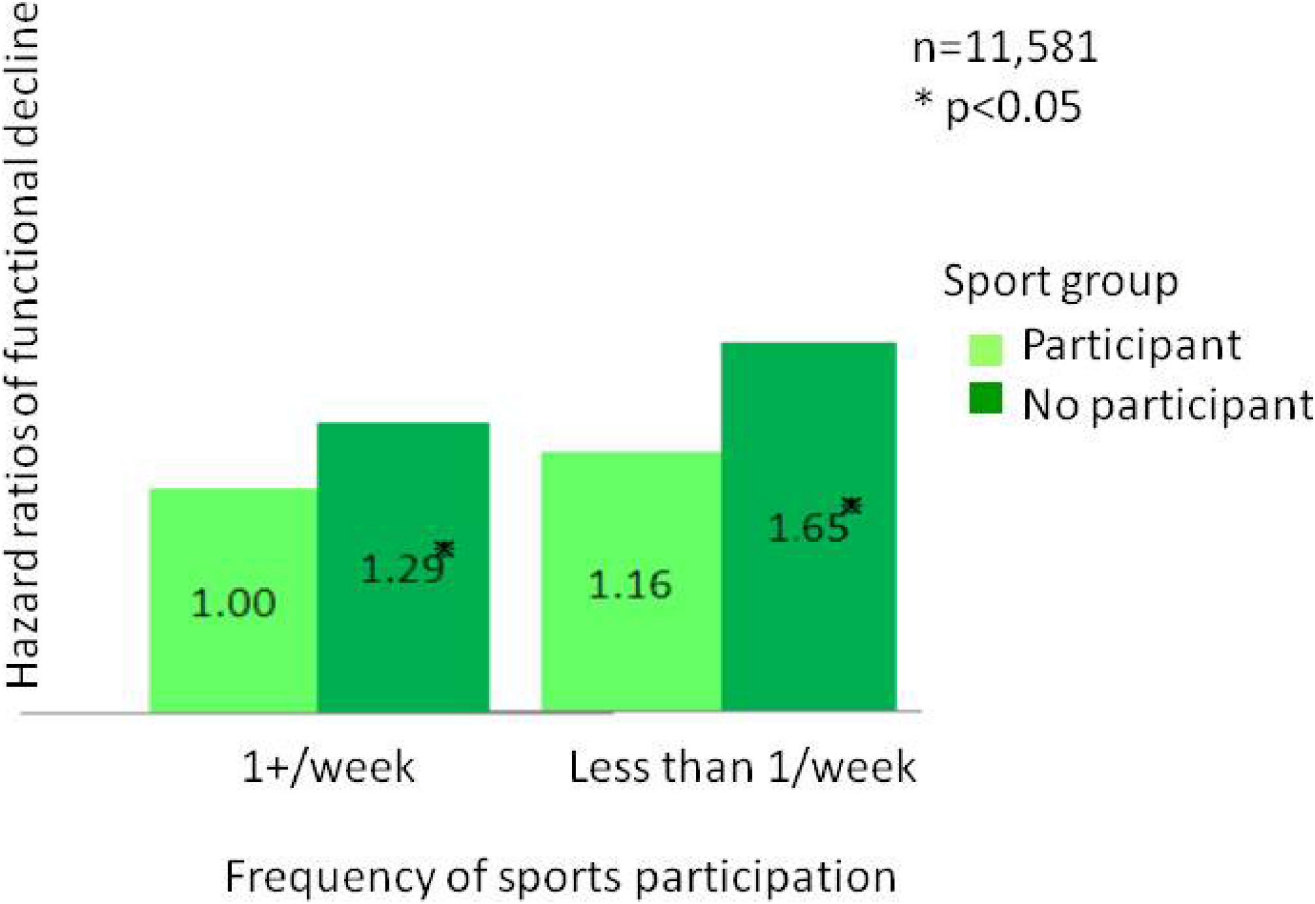
Hazard ratios of functional decline by frequency of sports participation: 4-year follow-up This figure shows the hazard ratios of functional decline in sports group participants by frequency of participation in sports. Hazard ratios were calculated after 4-year follow-up and are adjusted for age, sex, annual equivalent income, educational attainment, marital status, occupation status, self-reported medical conditions, depression, smoking, alcohol consumption, and exercise. Reference: Kanamori S, Kai Y, Kondo K, Hirai H, Ichida Y, Suzuki K, Kawachi I. Participation in sports organizations and the prevention of functional disability in older Japanese: the AGES Cohort Study. PLoS One. 2012. http://www.plosone.org/article/info%3Adoi%2F10.1371%2Fjournal.pone.0051061

Most interestingly, an analysis of participation rates in sports clubs and rates of functional decline found that the two factors are negatively correlated (Figure 2; *r* = −0.6, *P* < 0.01). As these were results of a cross-sectional study, they may easily be dismissed as the product of reverse causation. However, as JAGES also conducted longitudinal studies, it was possible to confirm that participation in more organizations tended to be associated with lower risks of functional decline.^5^ Hazard ratios of functional decline by the number of groups in which subjects participated were 0.83 for 1 group, 0.72 for 2 groups, and 0.57 for 3 groups compared to non-participation (Figure 3). We also found that, among those who participated in sports once or more per week, people who played sports alone showed significantly higher hazard ratio (1.29) of functional decline compared to those who played sports in a sports group (Figure 4).^6^

## THE NEED FOR COMMUNITY PERSPECTIVE

Past research has mainly focused on an individual or high-risk approach, which tries to prevent health problems by finding high-risk individuals to target for education and intervention. Such secondary prevention programs have been in place since 2006 under the public long-term care insurance in Japan. However, these programs have not been successful.

The findings above suggest that, in addition to programs focused on high-risk individuals, there is a need for a community perspective. By focusing on the community and endorsing participation in social organizations, older people may form social networks that can provide social support, which is known to be a positive factor for maintaining health.^7^ Since April 2015, the Japanese government has enlarged the target of preventive policy from secondary prevention policy focused to including primary prevention policy based on the community approach suggested by the findings of JAGES.

## JAGES HEART

The JAGES HEART is a tool to assist in implementing a community approach to preventive policy.^8^ JAGES HEART is a benchmark system for the population’s health that was developed based on Urban HEART,^9^ a management tool developed by WHO Kobe Center for all ages and types of countries (including developing countries). The Urban HEART framework was adapted for an older population and for use in Japan and was named JAGES HEART. JAGES HEART is also designed to provide indicators for age-friendly cities (AFC).^10^

WHO suggests a four-stage management cycle, including assessment, response, policy, and program stages.^9^ In the assessment stage, the problem is defined, and in the response stage, the agenda is set. Then, in the policy stage, a policy is developed, which is implemented in the program stage. After the program is implemented, monitoring and evaluation takes place, and the program is assessed again to complete the cycle.^9^ JAGES HEART is applicable to all stages of this management cycle.

JAGES HEART is funded by the Ministry of Health, Labour and Welfare and benchmarks indicators of the health and community environments, particularly for prevention of functional decline, using multi-faceted indicators derived from the JAGES dataset. Analysis of the JAGES survey partially revealed six core areas that are important for monitoring health status of the community: summary indicators, specific indicators, physical environment, human and social development, economy, and governance. Summary indicators include all-cause mortality, proportion of people eligible for long-term care, proportion of new certifications for long-term care requirement, proportion of people with high quality of life, and self-rated health. Specific indicators include cause-specific mortality, rate of response to the basic checklist developed by the Ministry, number of remaining teeth, low body mass index, and depression. Physical environment includes parks or roads suitable for walking and number of falls in a year. Human and social development includes the proportion of respondents having health check-ups over the preceding year, proportion of people with smoking habits, walking time, number of “shut-in” older individuals, proportion of respondents participating in sports clubs, proportion of respondents participating in volunteer activities, and number of projects for social participation, such as “salons” (community center programs). Economic indicators include average taxable income and proportion of respondents receiving welfare benefits. Governance indicators include budget amounts for projects to prevent the need for long-term care (per older individual) and long-term care premium (by income class).

**Figure 5.**
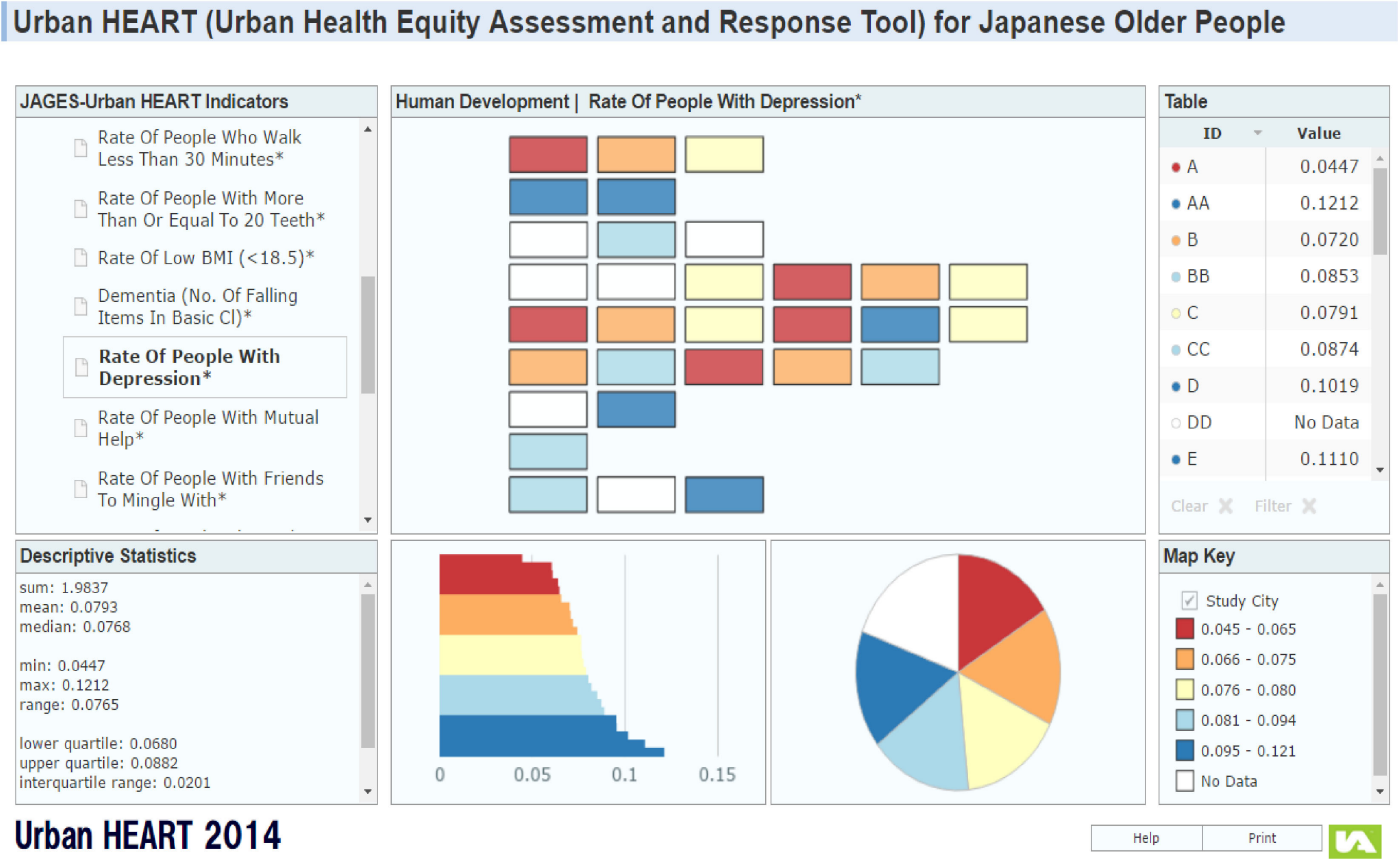
Urban HEART for Japanese Older People Reference: http://sdh.umin.jp/heart/Single_map.html

A visible format was developed in order to make the results easier to understand. For example, Figure 5 shows the prevalence of depression among 31 municipalities from the JAGES 2013 survey. This format makes it easy to see which municipalities show higher prevalence of depression (and therefore, higher risk of low social participations). Based on these results, civil servants in the high prevalence municipalities may set targets for addressing the indicators.

**Figure 6.**
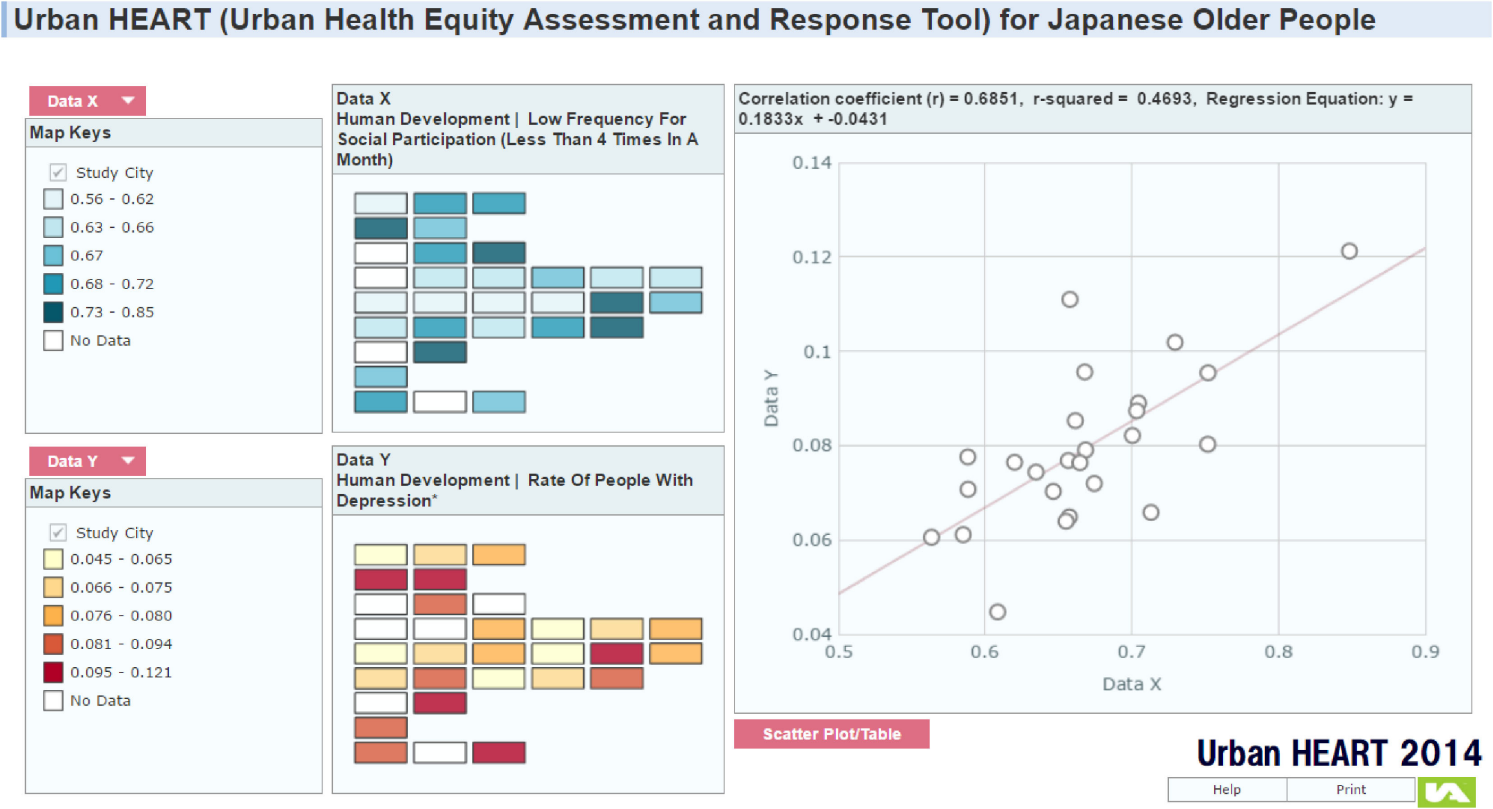
Correlation between rate of low social participation and rate of depression Reference: http://sdh.umin.jp/heart/Double_map.html

The relationships among indicators have also been analyzed. For example, the lower frequency of social participation was found to positively correlate with the prevalence of depression (Figure 6; *r* = 0.69). The graphical information or mapping system was used on different levels of community, and it was observed that when analysis was done at the school district level, the same findings were repeatedly made. Such information suggests that facilitating social participation may have a positive impact on the population health of older people.

A similar relationship was found between standardized mortality rates and social participation. A follow-up study in municipalities (*n* = 23) showed that municipalities that successfully increased prevalence of those who walked 30 minutes or more per day tended to have decreased fall rates, implying a causal relationship that promoting walking or social participation results in better health status of older people.^11^ Thus, JAGES HEART is regarded as a useful tool to assess and respond to the questions: “what is the problem?” and “which kind of intervention are needed and effective for the community agenda?”.

## IMPLICATIONS OF THE TWO STUDIES

Two important implications can be derived from these two projects. First, both projects point to the fact that social participation is a significant determinant of health in older people. This is good news, as promoting social participation is relatively easy and can be adopted by developing countries as well as developed countries. Second, a community approach might be useful for managing health policy and public health practice, and JAGES HEART provides a useful benchmarking system for this purpose.
